# Experimental evidence on improving COVID-19 vaccine outreach among migrant communities on social media

**DOI:** 10.1038/s41598-022-20340-2

**Published:** 2022-09-28

**Authors:** Jasper Tjaden, Esther Haarmann, Nicolai Savaskan

**Affiliations:** 1 Department of Economic and Social Sciences, University of Potsdam, Potsdam, Germany; 2 International Organization for Migration, Global Migration Data Analysis Centre, Berlin, Germany; 3 Department of Public Health Neukölln, District Office Neukölln of Berlin, Blaschkoallee 32, 12359, Berlin, Germany

**Keywords:** Health care economics, Public health

## Abstract

Studies from several countries suggest that COVID-19 vaccination rates are lower among migrants compared to the general population. Urgent calls have been made to improve vaccine outreach to migrants, however, there is limited evidence on effective approaches, especially using social media. We assessed a targeted, low-cost, Facebook campaign disseminating COVID-19 vaccine information among Arabic, Turkish and Russian speakers in Germany (N = 888,994). As part of the campaign, we conducted two randomized, online experiments to assess the impact of the advertisement (1) language and (2) depicted messenger (government authority, religious leader, doctor or family). Key outcomes included reach, click-through rates, conversion rates and cost-effectiveness. Within 29 days, the campaign reached 890 thousand Facebook users. On average, 2.3 individuals accessed the advertised COVID-19 vaccination appointment tool for every euro spent on the campaign. Migrants were 2.4 (Arabic), 1.8 (Russian) and 1.2 (Turkish) times more likely to click on advertisements translated to their native language compared to German-language advertisements. Furthermore, findings showed that government representatives can be more successful in engaging migrants online compared to other messengers, despite common claims of lower trust in government institutions among migrants. This study highlights the potential of tailored, and translated, vaccination campaigns on social media for reaching migrants who may be left out by traditional media campaigns.

## Introduction

Studies have found that many migrant groups in high-income countries are at increased risk of COVID-19 infection^1^. This may be due to risk factors such as poor housing and employment conditions as well as barriers to healthcare affecting access to prevention and control measures such as vaccination. Lower COVID-19 vaccination rates among migrant communities compared to the general population have been reported in the US, UK and Germany^2–8^. In November 2021, approximately 11 months after the COVID-19 vaccine became available in Germany, the (self-reported) COVID-19 vaccination rate among persons in the country with a ‘migrant background’ (84%) was lower than among those without a ‘migrant background’ (92%) (see appendix, Sect. 1, for definition of migrants)^8^.

Lower vaccination rates may partially be a result of insufficient information regarding vaccine benefits and access. Recent survey evidence from Germany suggests that first and second-generation migrants are more likely to feel uncertain regarding facts about the COVID-19 vaccine and are more often misinformed^8^. Two crucial barriers to vaccine information which have been identified in the literature include language and trust barriers^2–11^.

First, recent migrants with lower levels of host country language acquisition may not consume mainstream media nor be able to access official information provided by health agencies and professionals. Official information and public vaccination campaigns are often not available in the dominant languages of migrants. Studies from Canada, Denmark and the US reported lags in translating official health guidance into foreign languages and poor dissemination to, and hence access by, migrant communities^10^. The lack of official communication translated in foreign languages was also seen in Germany. Germany has approximately four million residents—or 5% of its population—with limited German language skills including a large number of recent refugees from Arabic speaking countries^12^. In November 2021, many official websites providing COVID-19 vaccine information and appointment booking tools were not available, or only partially available, in Arabic, Turkish, Farsi, Polish and other languages of major migrant groups in Germany.

Second, migrants may be less responsive to official information from government authorities (even if it is provided in their language), given lower levels of trust in representatives from host country institutions. In many countries, migrants face a higher risk of social exclusion, marginalization, and discrimination than non-migrants^9,11^. In many high-income countries, migrants, on average, earn lower incomes, face poorer working conditions, and have lower educational attainment^13^. Migrants may mistrust government institutions because of the perception that they are being denied the support necessary to achieve greater social mobility. In addition, many migrants experience direct discrimination on the labour and housing market^14–16^. Based on negative experiences, migrants may associate government institutions and their employees as representatives of the ‘majority’ population who have been the perpetrators of discrimination in their daily lives. Some evidence, including from Germany, suggest that public authorities disadvantage migrants directly by responding more negatively to formal requests compared to requests filed by non-migrants^17^. Negative direct experiences with authorities may lead to mistrust in government as a whole, including health agencies, and, thus, reduce the willingness to consume health-related information provided by government authorities. Lastly, migrants, particularly refugees, from countries with more authoritarian or more corrupt governments may generally hold high levels of mistrust in government institutions as a result of negative experiences in their countries of origin.

As a result of both language and trust barriers, migrants may be more likely to rely on information from social media and general information shared within their ethnic networks, available in their native language and provided by trusted sources, compared to mainstream media in the host country language^18–20^. Previous studies have shown that misinformation is widespread on social media platforms^21^.

Experts have highlighted the need for improved outreach to migrants, including through social media, to lower barriers to COVID-19 vaccination, encourage uptake^10,18,22,23^, and deliver equitable access to vaccines^22,24^. Social media has become a pivotal communication tool for governments and organizations to disseminate public health information^16–19^. However, the evidence on different outreach approaches is limited, especially among harder-to-reach migrant groups^25–27^.

In this study, we evaluate a targeted social media campaign on Facebook disseminating COVID-19 vaccine information among migrant groups in Germany. Advertisements encouraged vaccination, provided easy access to official COVID-19 vaccination appointment booking tools, and access to general information about the vaccine (see Fig. 2). We disseminated advertisements to Arabic, Turkish, and Russian speakers in November and December of 2021. Focusing on potential key barriers to vaccine information, we provide experimental evidence on the effects of native vs host country language advertisements and on the effects of different messengers depicted in the advertisements on responsiveness to the advertisements.

Overall, we aim to provide insights into how public health agencies can leverage online outreach to enhance vaccine information and access among migrant groups.

## Results

### Campaign reach

**Figure 1:**
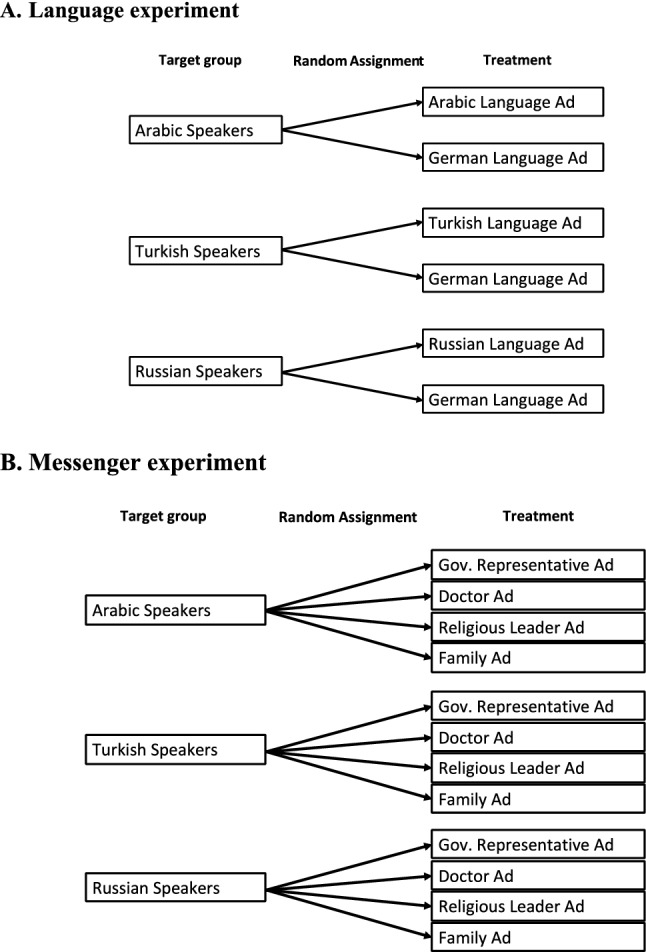
Experimental design. *Note*: Samples were first collected in Berlin (November 2021) and then scaled up to all of Germany (December 2021). For the Berlin sample, only the Arabic speakers were targeted.

**Figure 2:**
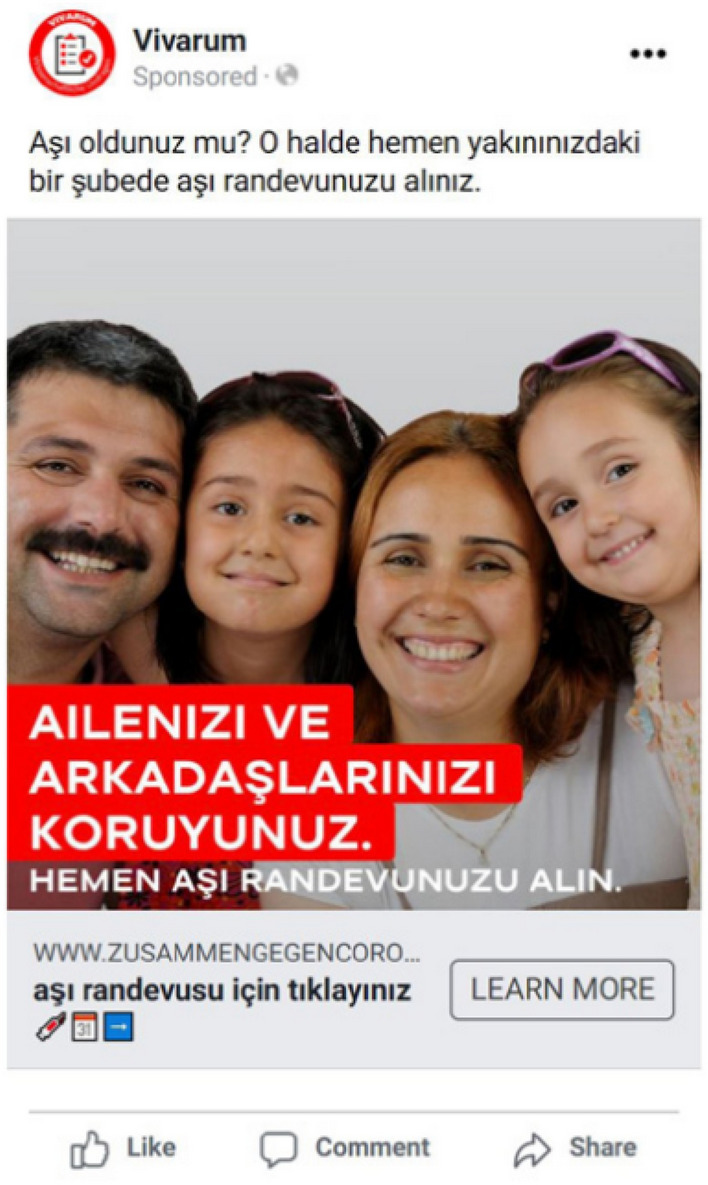
Example of Facebook advertisement used in the online COVID-19 vaccination campaign (in Turkish). *Note:* Vivarum Facebook Ad Manager, 2022; English Translation—Top: “Are you vaccinated yet? Now you can easily book a vaccination appointment close to you.” In image: “Protect your family and friends. Book a vaccination appointment now.” Button: “Get the vaccination appointment here.”

In total, the campaign included 36 separate advertisements, including nine in Berlin and 27 in all of Germany (see Figs. 1 and 2 in the methods section below). It ran for a total of 29 days, including 16 in Berlin and 13 in all of Germany (without Berlin). All advertisements combined were seen by 888,994 unique Facebook users (i.e. “reach”; see Table 1), including 108,829 users in Berlin and 780,165 users in Germany (without Berlin) and averaging approximately 30,654 users per day. Users saw the targeted advertisements, on average, two times. The campaign was more successful in reaching male users between the ages 25 and 55 years than female users and users under 25 as well as over 55 (see appendix, Table S1, for breakdown by sub-groups).

**Table 1:** Online campaign reach relative to Facebook population in November and December 2021.

Sample location	Target language group	Estimated total # of facebook users*	# reached by campaign	Reached by campaign (%)	# of clicks on advertisements	Click rate among those reached (%)
Berlin	Arabic	113,200	108,829	96	2479	2.3
Germany	Arabic	1,200,000	337,088	28	6495	1.9
	Turkish	1,400,000	278,157	19	3693	1.3
	Russian	638,300	164,920	26	2391	1.4
Total	All	3,351,500	888,994	27	15,058	1.7

Note: Data collected by the authors. *As of November 2021.

Overall, in Berlin and Germany combined, the campaign reached 445,917 Arabic speakers, 278,157 Turkish speakers, and 164,920 Russian speakers. In Berlin, the campaign reached 96% of Arabic speaking Facebook users; whereas in Germany, the campaign reached 28.1% of Arabic speakers, 19.9% of Turkish speakers, and 25.8% of Russian speakers on Facebook (see Table 1).

### Click-through rates

Across all advertisements, 15,058 (or 1.7%) out of 888,994 total exposed users clicked on the COVID-19 vaccine advertisement. This is equivalent to a click-through rate of 16.9 out of 1000 users. In Berlin, 2,479 (2.3%) users clicked on the advertisement and, in Germany, 12,579 (1.6%) users clicked on the advertisement. These click-through rates are higher than average industry benchmarks for comparable health care related campaigns on Facebook (0.83%)^28^. The click-through rate was comparable across age groups as well as across male and female users (appendix, Table S1). The click rate was higher among Arabic-speaking users (2.3% in Berlin; 1.9% in Germany) compared to Turkish speakers (1.3%), and Russian speakers (1.4%) (see Table 1). The difference in click rates between Arabic speakers and all other groups in Germany is statistically significant (*p* < 0.001).

### Extrapolated conversion rates

Among those users in Berlin who clicked on the advertisement, 1328 (53.6%) users visited the final vaccine booking tool linked in the Facebook advertisement. If this proportion is applied to the sample from all of Germany, 6740 would have visited the final vaccine tool. Assuming 10% of individuals who visited the vaccine booking tool eventually received a vaccination (i.e. a benchmark conversation rate for health campaigns, see appendix, Sect. 5), advertisements in Berlin and Germany would have potentially resulted in an estimated 807 vaccinations (28 per day). Assuming 20% of individuals who visited the vaccine booking tool eventually received a vaccination, advertisements in Berlin and Germany would have potentially resulted in an estimated 1614 vaccinations (56 per day) (appendix, Sect. 5, for details on extrapolation).

### Cost effectiveness

The total (non-staff) cost for the 29-day campaign was 6,455 EUR, 0.007€ per person reached by an advertisement. The cost per person who accessed the vaccination appointment tool linked in the advertisement was 0.43€ per person, equivalent to 2.3 individuals for every one euro spent on the campaign. Dividing the costs for the advertisements by the number of assumed vaccinations (i.e. from estimated conversation rates) yields a per-vaccination cost of 8 EUR for the 10% scenario and 4 EUR for the 20% scenario (see appendix, Table S2).

The campaign cost was slightly higher for older users above 55 years as well as for female users compared to male users. The cost also varied by language group. For example, the cost to engage Russian speakers was almost three times as high compared to the cost for Arabic speakers (appendix, Table S2).

### The effect of native-language outreach

**Figure 3:**
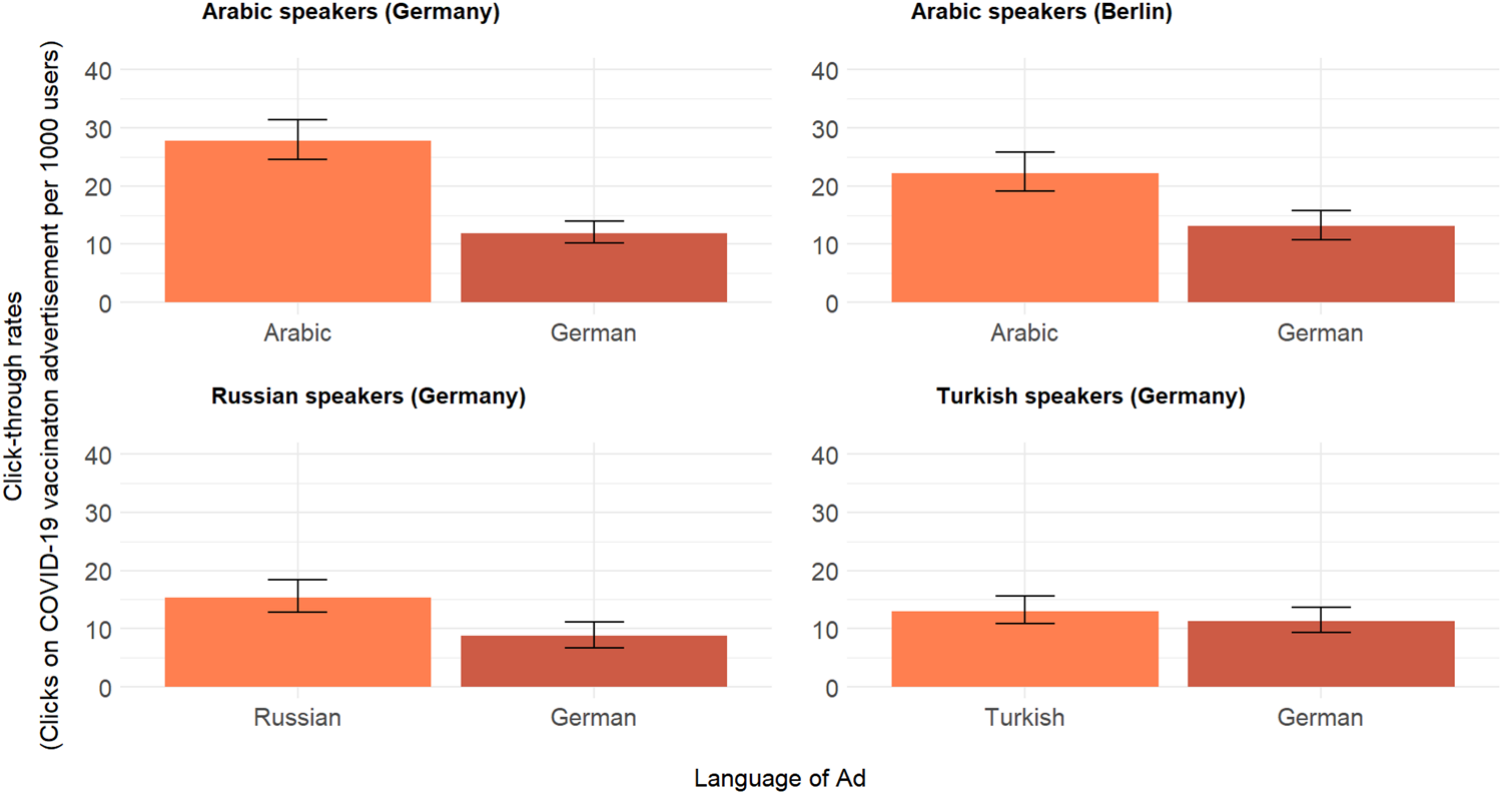
Clicks on COVID-19 vaccine advertisement per 1000 Facebook users by campaign location and language group, November and December 2021. Note: Pearsons chi-squared test yields significant difference in click-through rates between German and translated ads (*p* < 0.001) for Arabic and Russian speakers. Differences for Turkish speakers are not statistically significant (*p* > 0.05).

Compared to the German-only COVID-19 vaccination advertisements, significantly higher click-through rates were seen with vaccine advertisements translated to Arabic (*p* < 0.001) and Russian (*p* < 0.001) among these respective language user groups. There was less of a difference between German and Turkish language advertisements (*p* = 0.315; see Fig. 3) for the Turkish speaking group. In Germany, Arabic- and Russian-speakers were 2.4 (95% confidence interval [CI]: 1.9; 2.9) and 1.8 times (95% CI: 1.3; 2.4) more likely to click on vaccine advertisement in their respective languages compared to German language advertisements (see Table 2).

**Table 2:** Logistic regression model assessing likelihood of clicking on COVID-19 vaccine advertisement by ad language and campaign location, November and December 2021.

Language of ad *(Ref.: German language)*	Berlin sample		Germany sample	
	*OR (95% CI)*	N	*OR (95% CI)*	N
Arabic vs. German	1.7*** (1.3; 2.2)	15,934	2.4*** (1.9; 2.9)	23,086
Russian vs German	n/a		1.8*** (1.3; 2.4)	14,984
Turkish vs German	n/a		1.2 (0.9; 1.5)	19,332

Note: Each cell based on separate weighted logistic regression model. Significance levels at *p < 0.10, **p < *0.05*, ***p < *0.01.*

### The effect of the messenger

**Figure 4:**
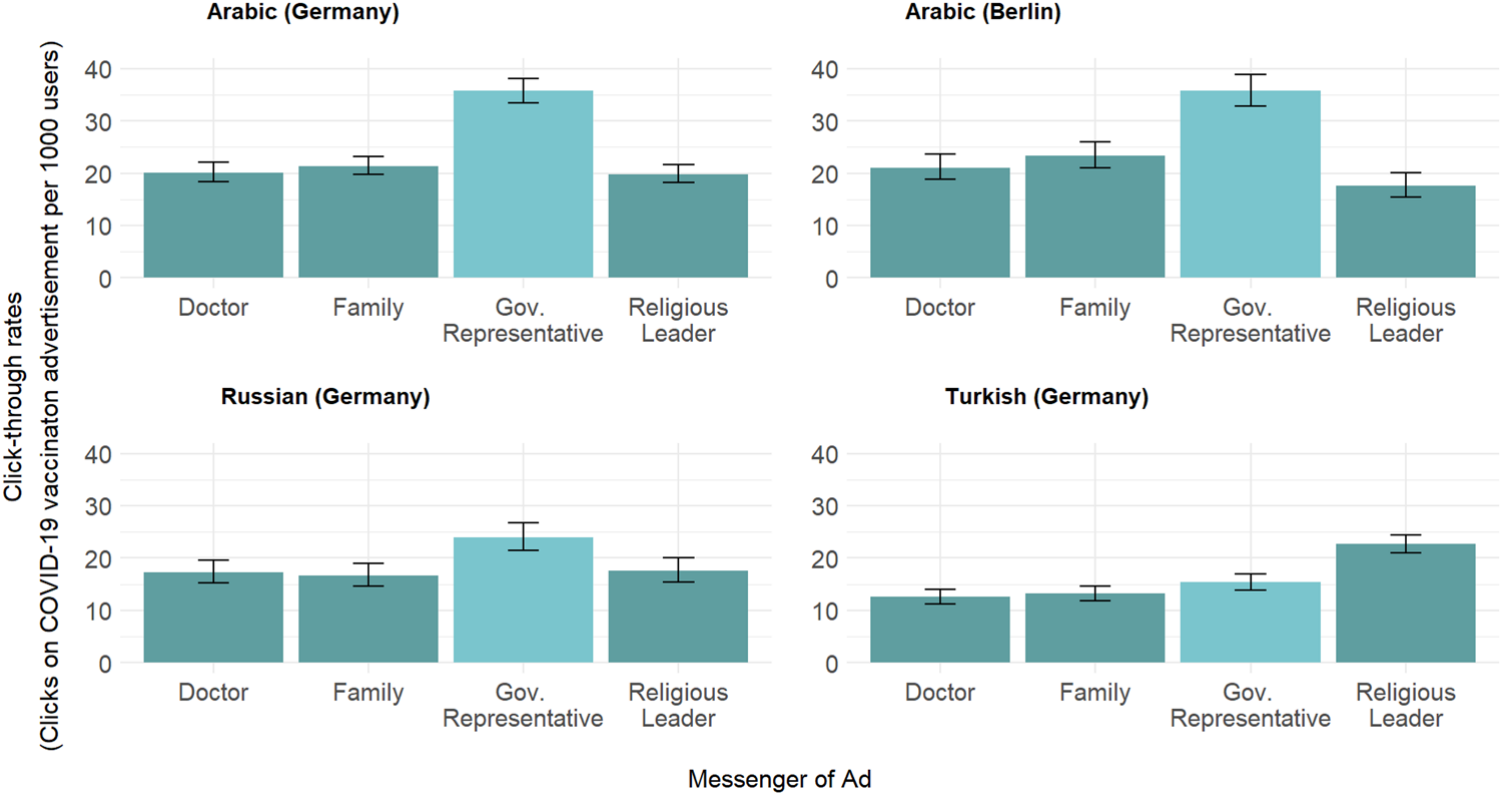
Clicks on COVID-19 vaccine advertisement per 1000 Facebook users by campaign location and image, November and December 2021. Note: Pearsons chi-squared test yields significant differences in click-through rates between government representatives and all other messengers (p < 0.001) among Arabic and Russian speakers. Among Turkish speakers, differences between religious leaders and all other groups is statistically significant at p < 0.001.

Compared to advertisements showing a doctor, a religious leader or a family, Arabic and Russian speakers were more likely to click on the advertisement depicting the government official (*p* < 0.001 for Arab speakers, *p* < 0.05 for Russian speakers; see Fig. 4). This pattern does not hold for Turkish speakers. Turkish speakers were more likely to click on advertisements depicting a religious leader compared to all other groups. In Germany, Arabic and Russian speakers were 0.5–0.7 times less likely to click on vaccination advertisements showing a doctor, a religious leader or a family compared to a government representative (see Table 3). Turkish speakers were 1.5 times more likely to click on the religious leader relative to the government representative (OR = 1.5, 95% CI; 1.3; 1.7, see Table 3).

**Table 3:** Logistic regression model assessing likelihood of clicking on COVID-19 vaccine advertisement by messenger and campaign location, November and December 2021.

Target group	Berlin sample		Germany sample	
	*OR (95% CI)*	*N*	*OR (95% CI)*	*N*
**Arabic speakers**				
Doctor vs. GR	0.6*** (0.5; 0.7)	56,303	0.6*** (0.5; 0.6)	100,288
Religious Leader vs. GR	0.5*** (0.4; 0.6)	56,303	0.5*** (0.5; 0.6)	100,288
Family vs. GR	0.6*** (0.6; 0.7)	56,303	0.6*** (0.5; 0.7)	100,288
**Russian speakers**				
Doctor vs. GR	n/a		0.7*** (0.6; 0.8)	53,592
Religious Leader vs GR	n/a		0.7*** (0.6; 0.93)	53,592
Family vs GR	n/a		0.7*** (0.6; 0.8)	53,592
**Turkish speakers**				
Doctor vs. GR	n/a		0.8* (0.7; 0.9)	103,304
Religious Leader vs. GR	n/a		1.5*** (1.3; 1.7)	103,304
Family vs. GR	n/a		0.9 (0.7; 1.0)	103,304

Note: GR = Government representative. All cells based on separate weighted logistic regression models. Significance levels at *p < 0.10, **p < 0.05, ***p < 0.01.

## Discussion

Urgent calls have been launched to provide equitable access to COVID-19 vaccines and accelerate the protection of populations by improving outreach to hard-to-reach groups such as migrants. However, the empirical evidence on effective strategies is limited, particularly regarding social media approaches. We conducted a unique, low-cost, randomised online experiment with a substantial sample size to assess the effects of a COVID-19 social media outreach intervention among migrants in Germany.

The results suggest that social media campaigns could be an effective, low-cost approach to providing migrants—a group with often lower vaccination rates and higher access barriers—with information about how to access vaccines. Within a period of 29 days, we reached 889,000 Facebook users, including 423,500 Arabic speakers, 268,000 Turkish speakers and 158,000 Russian speakers. Among those reached by the campaign advertisements, there was an overall click-through rate of 16.8 out of 1000 users (higher than comparable industry benchmarks). While this may appear low at first sight, it is important to consider that: (1) 68% of the German population was already fully vaccinated at the start of the study (as of 25 November 2021)^8^, and (2) some users may be generally hesitant to click on any advertisement on Facebook. The highest click rate was among Arabic-speakers. This could suggest a higher interest in vaccination among Arabic-speakers in Germany, a group that includes a large proportion of refugees who have more recently migrated to Germany from Syria, Iraq, and Afghanistan^29^.

For every euro spent on the campaign, 2.4 individuals accessed the vaccination appointment tool linked in the advertisement. Assuming that 10–20% of visitors to the vaccination appointment booking tool eventually received the vaccine, the estimated cost of the campaign would range from 4 to 8€ per vaccination.

National institutions, local authorities and migrant outreach organizations may benefit from the ease with which a social media campaign can expose a large percentage of migrants to health communication quickly and at a low cost. Social media platforms allow campaigns to target specific audiences including by language and tailor messages to specific needs—a limitation of conventional radio, TV and billboard campaigns.

Results illustrated the large effect of translating outreach to the native language of migrants and the large extent to which sizable populations are excluded from health communication if governments do not improve translation of public health communication materials. The effect of native language outreach was especially large for Arabic speakers, a more recent immigrant group in Germany with limited German skills^26^. The translation effect was less pronounced for Turkish speakers, which may represent longer established migrant communities in Germany^27^. The Russian speaking group likely reflects a mix of a smaller group of recent migrants and a larger group of long-settled migrants (so-called ‘Spät-Aussiedler’) who may speak both Russian and German. Variation across groups highlights the importance of customizing campaigns to specific migrant communities and demographics.

The results also suggest that the use of certain messengers in COVID-19 vaccine online campaigns such as government representatives can increase the likelihood of engagement with the materials across different migrant groups. For example, we found that Arabic speakers were significantly more likely to click on vaccine advertisements with images of government representatives compared to advertisements depicting religious leaders, doctors or families. This finding contradicts common claims in the literature that migrants have lower levels of trust in authorities^2,6,7,11^. Interviews conducted with representatives from the migrant community in Berlin also indicated that trust in the German government may be high among groups such as Arabic speakers, often comprised of many recent refugees. This could be due to having received protection from the Government of Germany as refugees^26,27^ or higher public sector performance relative to origin countries. The same interpretation may apply to Russian speakers. Many Russian speakers may have migrated to Germany because of low trust in home authorities and, thus, have higher trust in German authorities. However, this same effect of government official messengers in campaign materials did not hold true for Turkish speakers. We hypothesize that most members of this group grew up in Germany, but, given a potential degree of social marginalisation and potential negative experiences with authorities, may have lower trust in government. In addition, it is possible that those Facebook users who still use Turkish as their profile language are older and more marginalized compared to Turkish migrants who have acquired German language skills.

Certain study limitations should be considered. First, even though Facebook has a broad userbase and the platform allows campaigns to be targeted to specific audiences, certain demographic groups may have been left out, including children and teenagers under 18 years and adults over 65 years, as well populations without internet access. Public authorities should be aware of such selective coverage when considering target groups and social media platforms for campaigns. Second, our evaluation of the COVID-19 vaccine advertisements did not directly measure the number of administered vaccinations due to the logistical challenge of tracking actual vaccinations. It is possible that users engage with the vaccine advertisement without the intention of getting vaccinated (i.e., curiosity), including users that are already vaccinated. However, engaging with vaccine advertisements still ensures the spread of reliable information which may be further shared within users’ social network. Fourth, we created a profile on Facebook and produced our own vaccine booking website (for the Berlin sample) to be able to target advertisements and track websites visits. To avoid any partiality or bias in how the campaign is perceived, we created a new and neutral profile. Naturally, however, the new brand we created did not enjoy any brand recognition or trust among Facebook users. It is likely that known and trusted actors, particularly health authorities, would have attracted even more engagement using a similar campaign, further increasing potential cost-effectiveness.

Overall, we hope to contribute a unique empirical account of a COVID-19 vaccine outreach campaign targeting migrants on social media. Our methodology of leveraging digital platforms to collect evidence on vaccine outreach is scalable to other countries and migration contexts and can be cost-effective as well as rigorous in terms of causal identification. Therefore, we encourage future research to leverage this approach to provide comparative evidence on how variation in outreach on social media, including the source account, the content, and the targeting strategy, can improve access to vaccine information among hard-to-reach groups.

## Methods

### Study design

The study was designed as an online experiment using Facebook as the delivery platform (see appendix, Sect. 4, for details on ‘digital trials’)^30^. Various COVID-19 advertisements were compared, leveraging the platform’s AB testing (i.e. split testing) functionality which allows campaign implementers to experimentally test the effectiveness of advertisements against each other through the double-blind, parallel random assignment of users to the respective advertisements.

### Participants

Two rounds of testing were conducted. In Berlin, eligible study participants included Arabic speaking Facebook users. In Germany, the testing was expanded to Arabic, Russian, and Turkish speakers. Arabic, Turkish and Russian represent three of the major languages of migrant groups in Germany (appendix, Sect. 1).

In 2020, there were 4 million households in Germany where German was not the dominant language^12^. There are currently 13.5 million first generation migrants living in Germany among which 4.8 million migrated within the last 10 years^12^. Since 2013, Germany has received a high number of asylum seekers, mainly from Arabic-speaking countries, including Syria, Iraq and Afghanistan^12^. Russian speakers represent a heterogenous group of long settled migrants (‘Aussiedler’), recent political refugees and labour migrants. Turkish speakers largely represent (descendants of) the long settled, post-war ‘guest-worker’ generation in Germany. According to the National Statistics Office, there are currently 1.73 million residents in Germany born in Arabic speaking countries, 1.34 million born in Turkey, 1.1 million born in the Russian Federation^12^.

In Germany, Facebook reports that 40–47 million people can be reached using Facebook’s advertisement platform, accounting for up to approximately 56.5% of the population^12^. Facebook estimates that, in Germany, there are approximately 5 million users who do not use German as their main language on Facebook. This is equal to 10.6% of all Facebook users in Germany and comparable to the share of first-generation migrants in the German population^12^.

Recruitment of participants is implemented automatically by the advertisement platform according to pre-selected user characteristics. For this study, different language groups were selected to reflect various migrant groups in Germany. Language groups were identified by the language in which users choose to use the Facebook interface in their settings or by the language they actively use on Facebook.

### Procedures

Included Facebook users were exposed to one of 36 COVID-19 advertisements using simple, double-blind randomization. In Berlin, advertisements were disseminated for 16 days (between 25 November 2021 and 23 December 2021). In Germany, advertisements were disseminated for 13 days (between 7 and 23 December 2021).

Randomisation is implemented automatically by the Facebook advertisement manager platform, providing a balanced composition of users across treatment arms^30^. The randomly assigned COVID-19 vaccine advertisement automatically appears on Facebook users’ feed when using the platform. Users consent to receiving advertisements when creating a Facebook user account.

First, users were randomly assigned to either a COVID-19 vaccine advertisement in their native language (i.e. Arabic, Turkish or Russian) or to the identical advertisement in German language (i.e. “language experiment”, see Fig. 1). The text in the ad and the picture are held constant.

Second, users were randomly assigned to advertisements displaying different images of messengers representing different authorities (i.e., government representative, religious leader or doctor) or an image of a family (i.e., messenger experiment; see Fig. 2). Ads were disseminated in the native tongue of each migrant group. The text in the ad was held constant. Different messengers were used to represent different authorities assumed to induce different levels of trust among migrants (appendix, Sect. 3)^2,6,7,11^. The government official is the main messenger of interest (see Introduction). The rationale for selecting religious leaders, doctors and families as comparison group is described in the appendix, Sect. 3. Images for the doctor, the religious leader and the family were adjusted for each language group (i.e., featuring an Orthodox-Christian priest for Russian speakers and an imam for Arabic and Turkish speakers). The government representative was the same across all language groups (appendix, Figure S1–S5, for all advertisements).

All advertisements contained a short text encouraging users to book their COVID-19 vaccination appointment (e.g., “Are you vaccinated yet? Now you can easily book a vaccination appointment close to you”). Each advertisement also included a slogan (e.g., “Protect your family and friends—book your vaccination appointment now”; see Fig. 2 and appendix, Figs. S1–S5). The aim of the advertisement was to encourage users to click on the provided link at the bottom of the advertisement which led them to a COVID-19 vaccination appointment booking tool/website with information (in their preferred language) on how to directly book a vaccination appointment online, via telephone or walk-in opportunities in their area as well as general vaccine information (appendix, Figs. S6, S7).

The design of the advertisements, including the choice of language and depicted images, was informed by a desk review of available COVID-19 vaccination information in Germany, key literature about vaccine uptake among migrant communities, and interviews with local stakeholders working with migrant communities in Berlin, including a local public health agency, affiliated social work providers and an agency for intercultural communication (appendix, Sect. 2).

### Outcomes

To study overall campaign effectiveness, we first assessed how many people were reached by the campaign in Berlin and Germany. *Reach* is defined by the number of unique Facebook users who have been exposed to a respective advertisement at least once on their feed.

Second, we assessed the rate at which exposed users clicked on the COVID-19 vaccination appointment link provided in the advertisement. This is considered the *click-through rate* which is defined as the number of unique users who click on the COVID-19 vaccination appointment link provided in the advertisement for every 1000 unique users who have been exposed to the advertisement at least once on their feed. Facebook automatically tracks engagement with advertisements. As such, common issues in medical trials such as compliance, item- or unit non-response, interviewer effects, or response biases do not apply.

Third, we extrapolated two scenarios of assumed *conversion rates* (i.e., 10% or 20% of individuals who visited the vaccine booking tool may eventually have received a vaccination) to estimate the number of potential vaccinations received resulting from our Facebook campaign (appendix, Sect. 5). The scenarios were selected based on education and healthcare industry benchmarks for conversion rates on Facebook^28^.

Fourth, we assessed *cost-effectiveness* of the campaign by calculating the cost per each person who engaged with the vaccine advertisement and the cost per each estimated COVID-19 vaccination (assuming 10–20% conversion rates). Costs included in the calculation were costs related to delivering the advertisements through the Facebook advertisement platform. Costs excluded staff time involved in developing the advertisements which may vary considerably across organizations. Depending on the scale of the campaign, designing an advertisement on Facebook may only necessitate a few hours.

Lastly, to measure the (causal) *effects* of the language and messenger experiment, we compared the *click-through rates* between advertisements. Aggregated data on reach and clicks for respective advertisement types, disaggregated by age, gender and language of the user, are provided by Facebook platform. No individual-level data is available.

### Statistical analysis

We descriptively summarised the aggregated data on reach and clicks by age, gender and language of users for the campaign in Berlin and Germany. We also described the overall number of potential vaccinations received according to the assumed conversation rate scenarios. Cost-effectiveness was estimated overall and according to the age, gender and language of the user.

In the language experiment, the click-through rates of advertisements in Arabic, Russian or Turkish were compared to those in German (i.e. reference group). In the messenger experiment, the click-through rates of advertisements depicting a government official (i.e. reference groups) were compared to those with a doctor, religious leader or family member. Pearson’s chi-squared test was used to test for statistically significant differences of click-through rates between groups (i.e., different advertisement designs by language and by messenger). Weighted logistic regression was also used to estimate the Odds Ratios (OR) for the likelihood of clicking on a specific advertisement over not clicking on it, according to language and messenger. These models were weighted by the number of those who saw the advertisement to account for aggregated data.

### Ethical considerations

No approval was required for this study. No individual-level data were collected, and all analyses included aggregated estimates only. The Facebook platform does not provide individual-level data according to data protection policies. Where applicable, the Declaration of Helsinki^31^ and CONSORT-EHEALTH statement (an extension of the CONSORT statement for improving reports of Web-based interventions) were followed^32^.

## Supplementary Information

Supplementary Information.

## Data Availability

Data and code can be shared upon request. Please contact Jasper Tjaden at jasper.tjaden@uni-potsdam.de.

## Data protection

Persons shown in Fig. 2 and Figures S1-S5 are not subjects of this study. The images were part of the intervention material. Images were acquired through the stockphoto platform canva.com. Under the terms of the user agreement, canva.com grants its users a perpetual, royalty-free, non-exclusive, worldwide license to use images exported from the platform. Persons shown in the images were photographed by professional photographers requiring prior consent. More information available at https://www.canva.com/policies/content-license-agreement/.
