# Supplementary material for: Experimental evidence on improving COVID-19 vaccine outreach among migrant communities on social media
Source: Sci Rep. 2022 Sep 28;12:16256. doi: 10.1038/s41598-022-20340-2 (PMC9518941; doi:10.1038/s41598-022-20340-2)
Supplement: Supplementary file 1 — Supplementary Information. [file 41598_2022_20340_MOESM1_ESM.docx]

**Supplementary appendix**

*Experimental evidence on improving COVID-19 vaccine outreach*

*among migrant communities on social media*

1. **Defining migration status**

The definition of a ‘migrant’ varies across countries. In several European Union (EU) countries, including Germany, a ‘migrant background’ refers to individuals who were born in another country (first generation) *or* who have at least one parent who was born in another country (second generation). Many other countries only consider first-generation migrants – those who were born abroad and migrated – as migrants. Aslyum seekers are migrants who claim international protection in another country based on grounds of persecution in their origin country. Refugees are migrants whose asylum claim has been approved.

In this study, due to empirical limitations, migrants are identified by the language they speak. Unfortunately, this is likely to largely capture first-generation migrants with limited destination-country language skills and more likely to exclude second-generation migrants and those with fluency in the destination-country language.

For certain origin groups, such as Arabic speakers, it is highly likely that many Facebook users are recent asylum seekers and refugees. Germany received over a million asylum seekers from Syria, Afghanistan and Iraq since 2013 (1).

1. **Preparatory desk review and qualitative interviews in Berlin**

In order to design the Facebook posts advertising COVID-19 vaccination, we first conducted a rapid desk review of online information available to migrant groups in Berlin, Germany. We found that many websites and vaccine booking tools (such as doctolib.de – the main tool used in Berlin) were only available in German (November 2021). At times, summary information was translated partially into other languages (mostly Turkish, Arabic, Romanian; Bulgarian) and made available in PDF documents on hard-to-find locations on websites. Second, we conducted a series of interviews with local stakeholders in Berlin who engage with migrant communities including a local health agency, social work providers and an agency for intercultural communication. Experiences with social media outreach were limited, and activities were scattered, ad-hoc and largely not evaluated – partially due to funding and human resource constraints. These discussions underscored the potential utility of exploring the use of social media outreach to migrant groups and reaffirmed the need to focus the study on language and trust barriers.

1. **Rationale for selecting comparison groups in messenger experiment**

The messenger experiment compared the responsiveness of Facebook users to advertisements depicting different types of messengers. The main messenger of interest was a government official who represents a government authority. This messenger was selected to see whether it triggers lower responsiveness by migrants given (assumed) lower levels of trust in government authorities (2–5). As comparison groups, we selected other messengers representing other types of authorities including a religious leader, family member and doctor.

The religious leader comparison was selected based on literature suggesting that certain migrant groups in Europe are more religious, on average, and respect religious leaders as authority figures in their community (6, 7).

The family member comparison was selected based on literature suggesting that many migrant communities have greater levels of family orientation compared to non-migrants. For example, an analysis published by the Konrad Adenauer Foundation emphasized that the importance of family is especially high among migrant communities in Germany. This finding is supported by results from the 2017 microcensus showing that 64% of people with a migrant background live with families, while only 44% of the non-migrant population live with families (8).

The doctor comparison was selected as a comparable baseline across all language groups. Doctors are generally respected in all societies. An evaluation by the Institute for Nursing Science at the University of Bielefeld showed that doctors are seen as persons of respect by Russian and Turkish migrants in Germany and enjoy a high level of trust among their patients (9).

1. **Digital trials using Facebook as delivery platform**

This study used Facebook as a delivery platform for an online experiment, testing COVID-19 vaccine advertisements. Using Facebook as a delivery platform has certain advantages over other alternatives such as customized online experiments using enrolment by the researcher or company solutions such as MTurk (10) including 1) high external validity through large population coverage, the ability to target specific population sub-groups, and the fact that experiments occur in a natural online environment (unlike lab and Mturk experiments); and 2) high internal validity based on randomization of users to treatments (i.e., advertisements) and prevention of overlap between control group and treatment group exposure. Facebook is currently the largest social network globally with 2.8 billion active monthly users.

1) In Germany, Facebook reports that 40-47 million people can be reached using Facebook’s advertisement platform in Germany, accounting for up to approximately 56.5% of the population.^^[[1]](#footnote-1)^^ Facebook estimates that, in Germany, there are approximately 5 million users who do not speak German (10.6% of all Facebook users, which is comparable to the share of first-generation migrants in the German population). Compared to alternative experimental settings, Facebook experiments have higher external validity due to their ability to reach large subsamples of the general population (11). As Facebook experiments occur in a natural online setting, they possess superior ecological validity compared to both laboratory and MTurk experiments (10). The naturalistic settings and lack of incentives for participants also eliminate the risk of demand biases, speeding and cheating, which are traditionally high in MTurk experiments.

2) The Facebook advertisement platform produces high quality data in terms of internal validity. The Facebook advertisement platform allows for so-called A/B testing (split testing) which randomly assigns users to be exposed to different advertisements varying in their content, text, images, language or target group. Facebook requires its users to provide personal information in terms of age and gender, and tracks location, allowing researchers to target audience segments according to needs.

The downside of conducting online experiments on Facebook is that only few dependent and independent variables are available. The main dependent variables are based on how often users click on a given advertisement relative to another advertisement. Independent variables can be introduced by varying the design or content of the advertisements (or treatments). Advertisement may vary the images, texts, language of texts, target groups or videos while holding all other conditions constant.

To target advertisements via Facebook, we created a unique Facebook profile (i.e. “vivarum”, see appendix, Figure S6). The alias was chosen to be neutral, inconspicuous and credible. We chose to create a new account to avoid any potential bias arising from perceptions towards existing accounts.

1. **Extrapolating Covid-19 vaccinations (“conversion rates”)**

# Vaccinations were not observed directly but estimated in two steps. First, for the Berlin sample, we were able to collect data tracking who visited our customized vaccine website.

Visitors arrive on the landing page (appendix, Figure S6) and can then choose to click on a subpage to enter the information on vaccination booking tools allowing users to book a COVID-19 vaccine appointment either via phone, online or through walk-ins. For the Berlin sample, 54% of users who clicked on one of the advertisements on Facebook eventually visited the vaccine appointment sub-page on our customized website. Unfortunately, it was not possible to track visitors for the Germany sample given that vaccine appointment tools vary considerably across regions. Consequently, in order to estimate the overall success rate, we applied the rate for Berlin (54%) to Germany samples. Lastly, we applied two scenarios regarding the percentage of sub-page visitors who have eventually been administered the vaccine: 10% and 20%. Marketing companies collect benchmark data on so-called conversion rates on Facebook, i.e. the number of people buying a certain product over the number of people who have been exposed to an advertisement about the product on Facebook. Benchmarks exist for various different industries such as “retail”, “auto”, “beauty” etc. Campaigns in the field of education are scored at a conversion rate of 13.6%, health care campaigns are scored at a conversion rate of 11% (12). We set the lower bound conversion rate for our health intervention at a comparable rate of 10%. However, we believe an upper bound (20%) scenario is warranted because, unlike most Facebook campaigns, our campaign did not market a for-profit product by a private company but a publicly available and potentially lifesaving good which was provided by the government (not private industry). At the height of the COVID-19 rollout, we believe that our campaign could have been more successful than benchmarks for for-profit campaigns would suggest. Therefore, we argue that a 10-20% range in our study provides a balanced and realistic assumption.

For interpretation of the conversion rate, consider the following example: 100 users click on the advertisement on Facebook, 54 continue to visit the vaccine appointment page on our website and, among those visitors, 5.4 users would be counted as being vaccinated in the 10% scenario, 11 users in the 20% scenario. A range from 10-20% is reasonable given that users already took several steps to arrive at the section of the website that links them directly to vaccine appointment booking tools, hence, signalling clear interest in the vaccine.

In Berlin, we created our own website which provided all necessary information. Using the customized website was not possible for disseminating the advertisements in all of Germany because vaccine information, booking tools and general access varies largely across regions. For the Germany sample, we linked to an official website by the federal government ([www.zusammengegencorona.de](http://www.zusammengegencorona.de), appendix, Figure S7), providing links to appointment booking tools for every region in addition to a federal telephone hotline. At the time of the study, the website was only available in Arabic, Turkish and Russian which therefore limited our choice of advertisement languages.

**Figure S1. Example of advertisements in the language experiment (Example German vs. Arabic)**


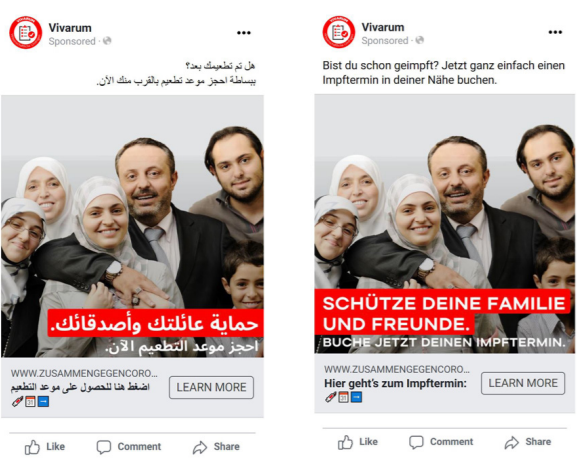


*Source:* Vivarum Facebook Ad Manager, 2022; The same test was also conducted in Russian and Turkish with varying images of families (see below)

**Figure S2. Example of advertisements in the trust experiment (Arabic speakers)**

| 1. Family   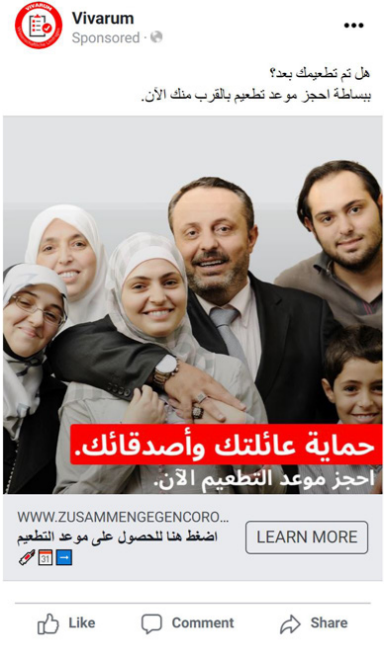 | 1. Government representative   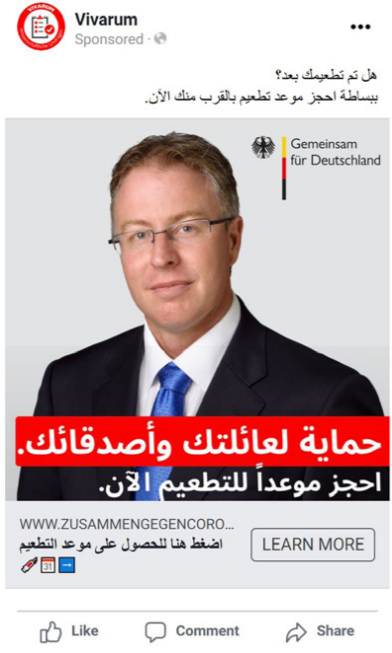 |
| --- | --- |
|  |  |
| 1. Religious leader   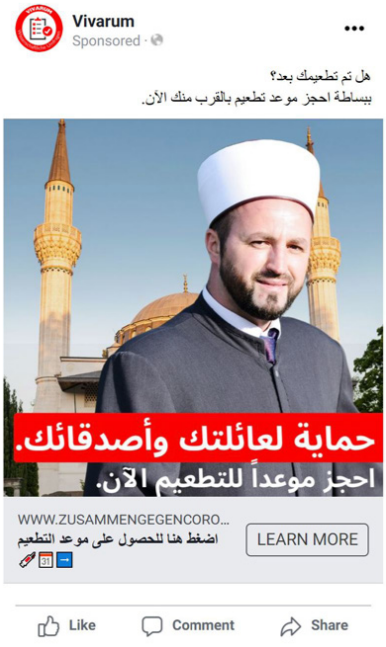 | 1. Doctor   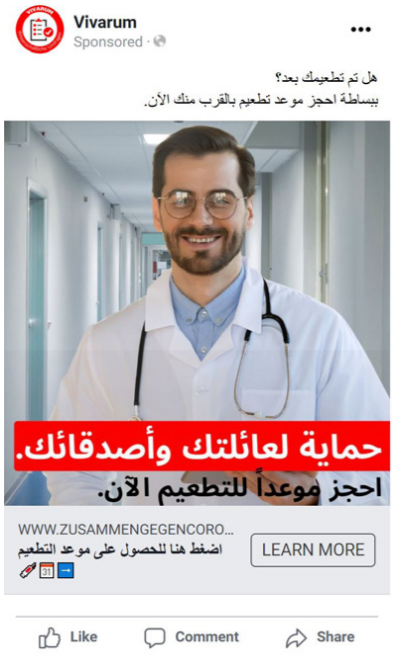 |

*Source:* Vivarum Facebook online experiment, November and December 2021

**Figure S3. Example of advertisements in the trust experiment (Russian speakers)**

| 1. Family   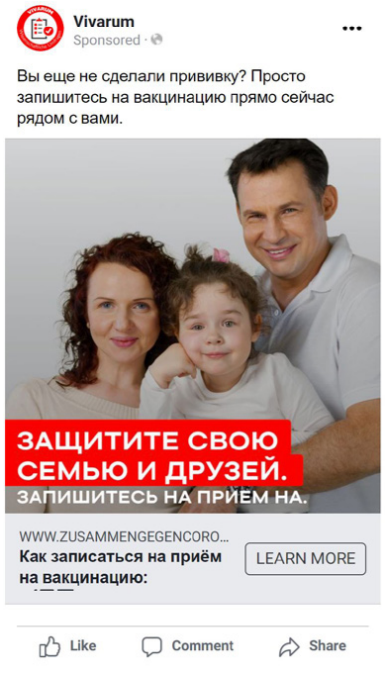 | 1. Government representative   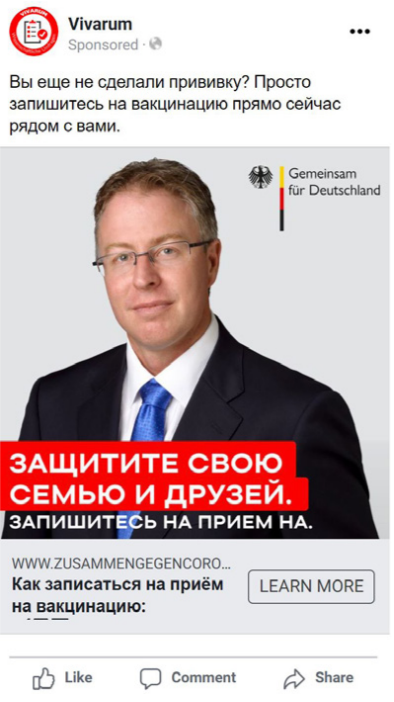 |
| --- | --- |
| 1. Religious leader   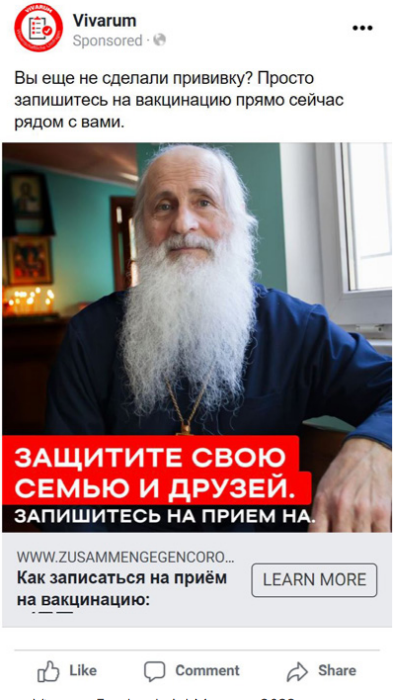 | 1. Doctor   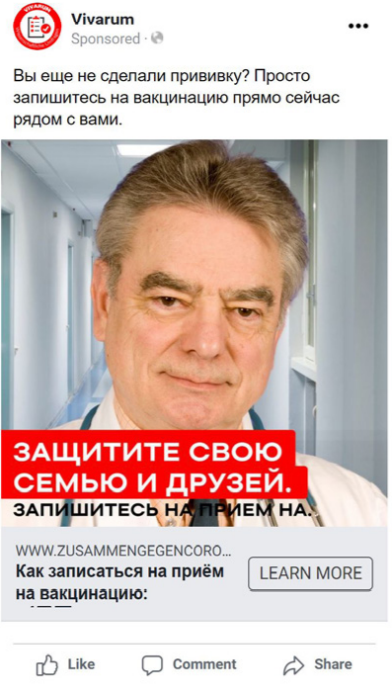 |

*Source:* Vivarum Facebook online experiment, November and December 2021

**Figure S4. Example of advertisements in the trust experiment (Turkish speakers)**

| 1. Family   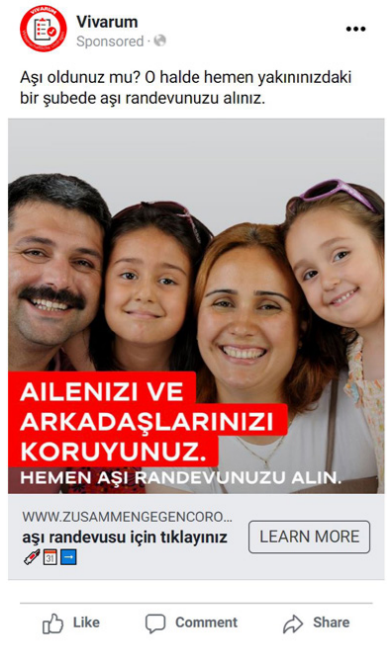 | 1. Government representative   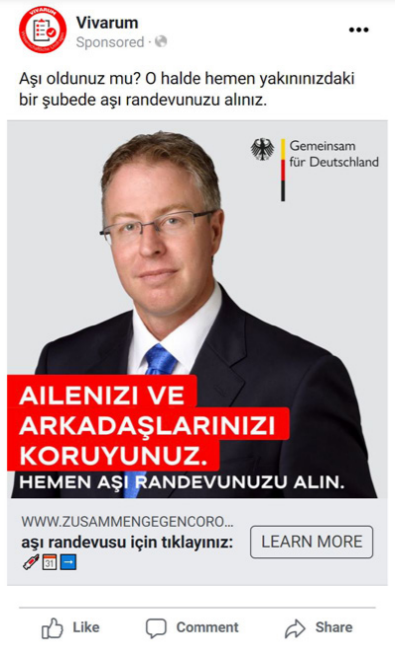 |
| --- | --- |
| 1. Religious leader   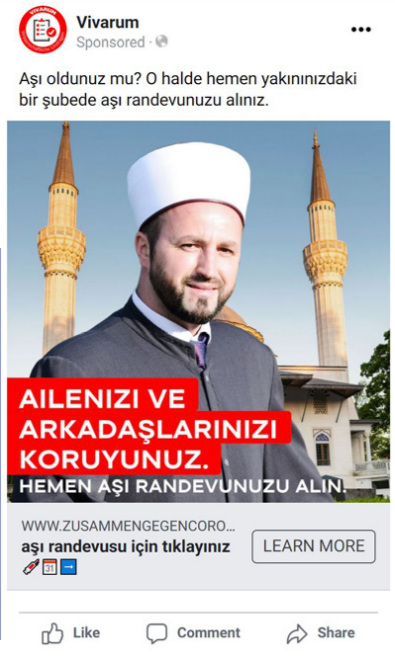 | 1. Doctor   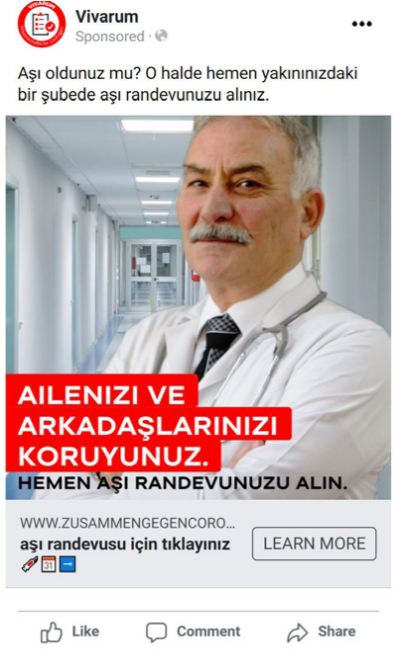 |

*Source:* Vivarum Facebook online experiment, November and December 2021

**Figure S5. Example of advertisements in the trust experiment (Russian speakers)**

| 1. Family   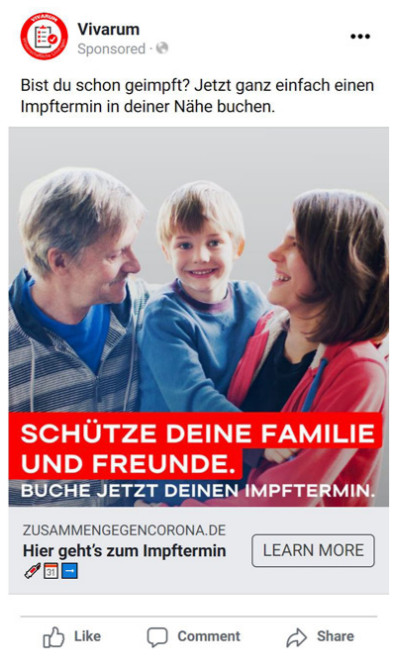 | 1. Government representative   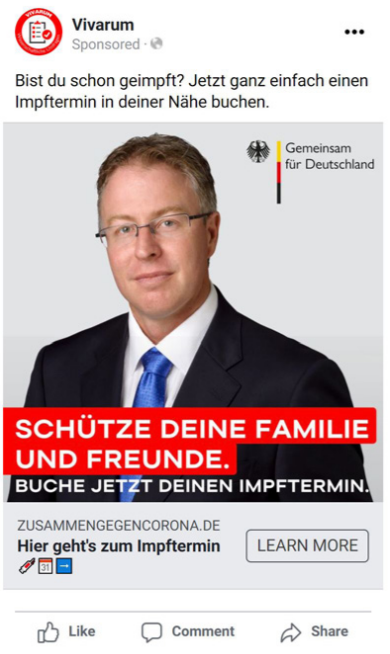 |
| --- | --- |
| 1. Religious leader   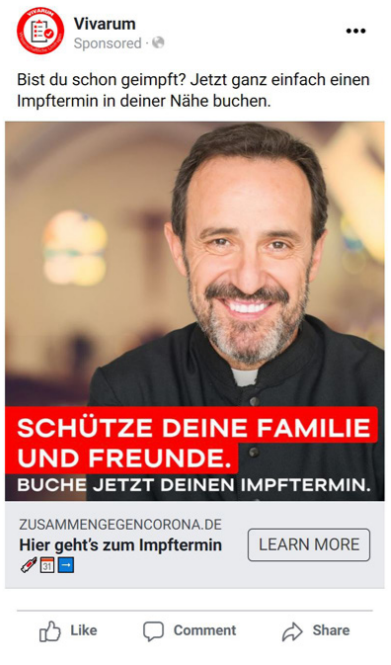 | 1. Doctor   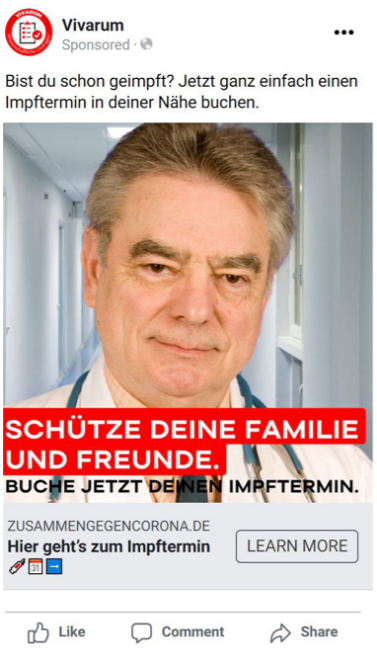 |

*Source:* Vivarum Facebook online experiment, November and December 2021

**Figure S6. Vivarum website created for Berlin sample (available in German and Arabic)**


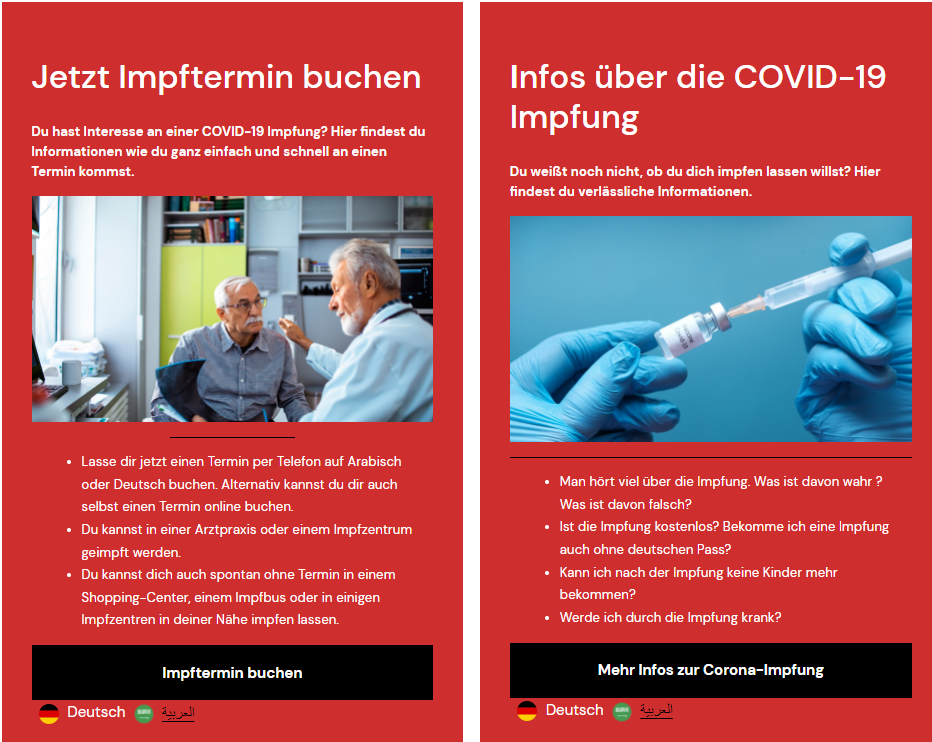

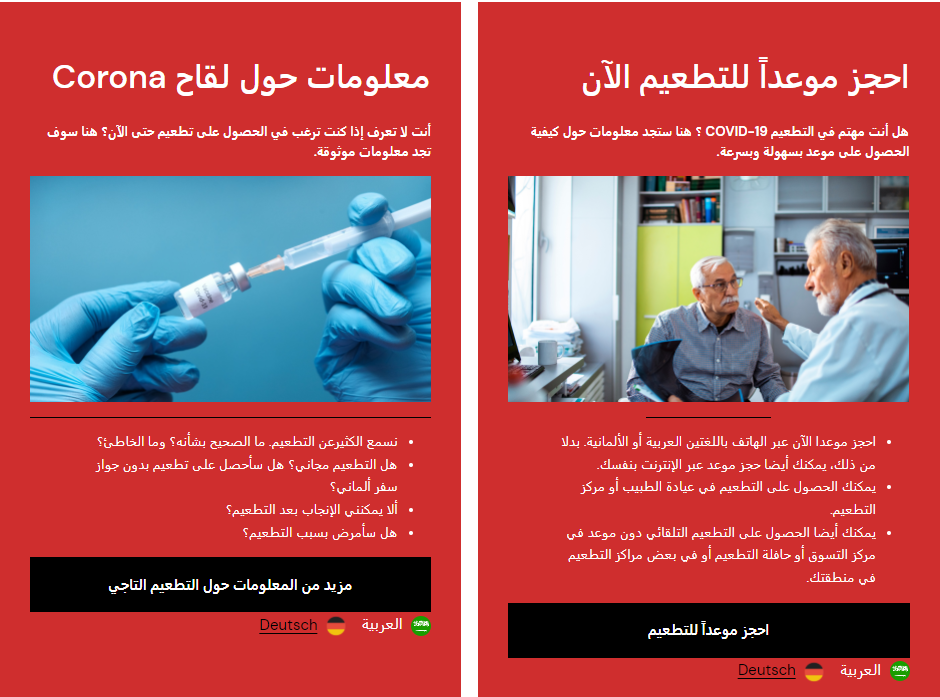


*Source:* Vivarum.org, 2022. This website was created by the authors to track appointment bookings in Berlin.

**Figure S7. ”Zusammen gegen Corona” – Website (in Arabic, Turkish, and Russian)**


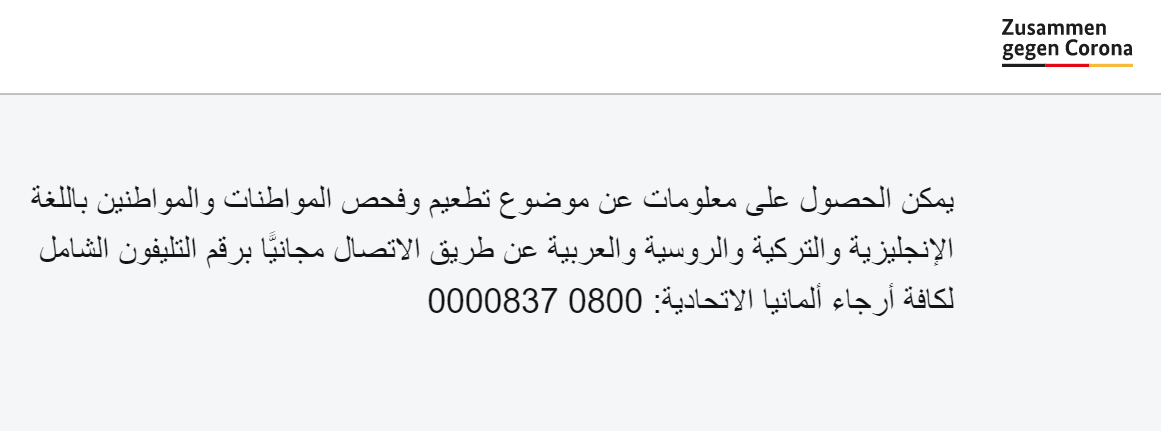

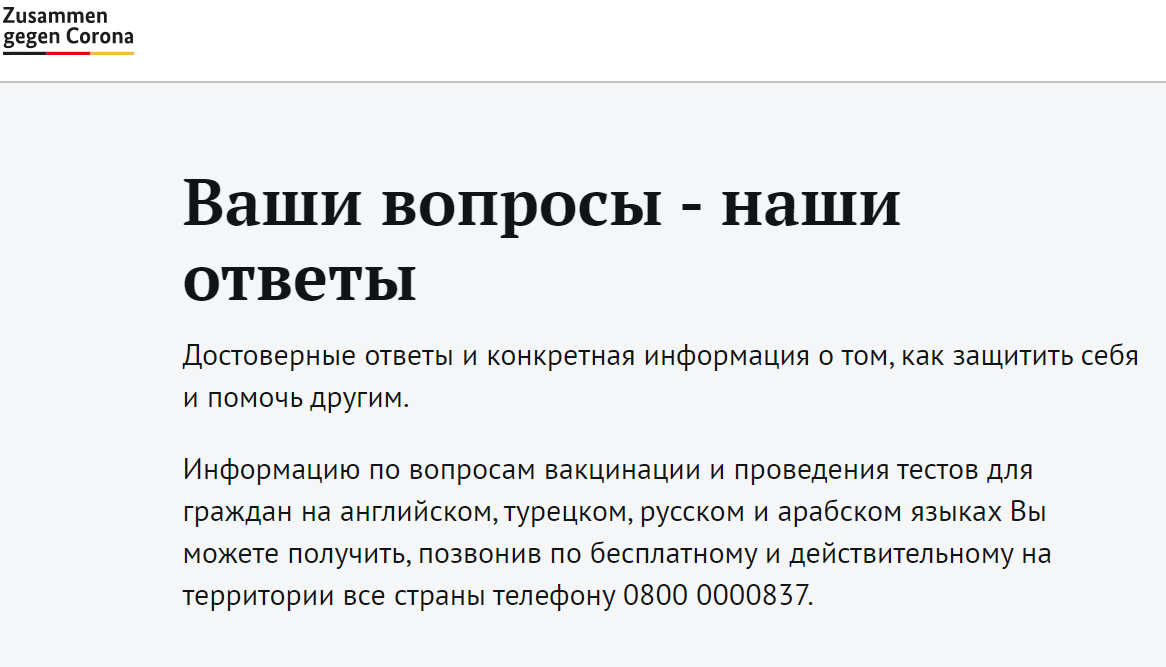

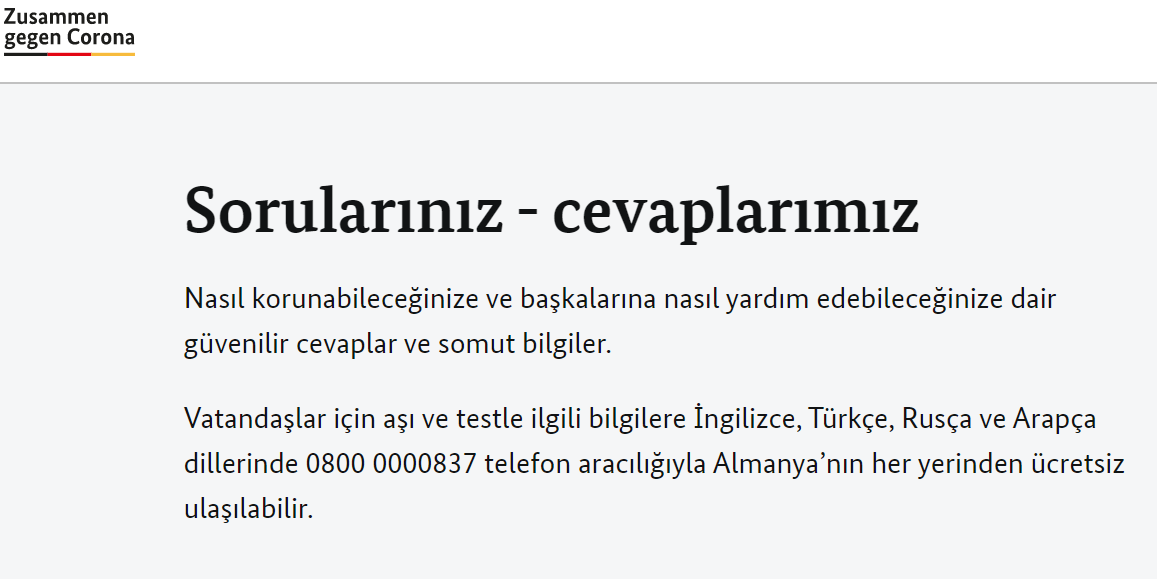


*Source:* German Federal Ministry of Health – zusammengegencorona.de, 2022. This website was used for the Germany sample.

**Table S1. Reach, spending and clicks by by sub-group**

| **Demographic** | **Group** | **Location** | **Total reach**  **(# users)** | **Spending (EUR)*** | **Unique clicks on ad** | **Click rate**  **(per 1000)** |
| --- | --- | --- | --- | --- | --- | --- |
| All | Total |  | 888,994 | 6,455 | 15,058 | 16.9 |
| Location | Location | Berlin | 108,829 | 1,669 | 2,479 | 22.8 |
|  | Location | Germany | 780,165 | 4,787 | 12,579 | 16.1 |
| Age | 18-24 | Berlin | 10,564 | 137 | 190 | 18.0 |
|  | 25-34 | Berlin | 40,688 | 570 | 824 | 20.3 |
|  | 35-44 | Berlin | 27,198 | 399 | 621 | 22.8 |
|  | 45-54 | Berlin | 16,568 | 283 | 420 | 25.4 |
|  | 55-64 | Berlin | 8,984 | 167 | 247 | 27.5 |
|  | 65+ | Berlin | 4,828 | 113 | 177 | 36.7 |
|  | 18-24 | Germany | 28,502 | 126 | 409 | 14.3 |
|  | 25-34 | Germany | 170,837 | 747 | 2,447 | 14.3 |
|  | 35-44 | Germany | 197,866 | 997 | 3,000 | 15.2 |
|  | 45-54 | Germany | 177,424 | 1,103 | 2,863 | 16.1 |
|  | 55-64 | Germany | 127,648 | 969 | 2,182 | 17.1 |
|  | 65+ | Germany | 77,888 | 844 | 1,678 | 21.5 |
| Sex | female | Berlin | 35,780 | 532 | 716 | 20.0 |
|  | male | Berlin | 72,693 | 1,130 | 1,753 | 24.1 |
|  | unknown | Berlin | 356 | 7 | 10 | 28.1 |
|  | female | Germany | 302,813 | 2,114 | 4,645 | 15.3 |
|  | male | Germany | 474,088 | 2,655 | 7,890 | 16.6 |
|  | unknown | Germany | 3,264 | 18 | 44 | 13.5 |
| Target Group | Arabic | Berlin | 108,829 | 1,669 | 2,479 | 22.8 |
|  | Arabic | Germany | 337,088 | 1,577 | 6,495 | 19.3 |
|  | Russian | Germany | 164,920 | 1,606 | 2,391 | 14.5 |
|  | Turkish | Germany | 278,157 | 1,604 | 3,693 | 13.3 |

Note: Data collected by authors. Statistics based on 36 individual advertisements, 9 in Berlin and 27 in all of Germany.

*Spending based on non-staff-related costs; breakdowns by location and group characteristic based on relative proportions.

**Table S2. Website visits, assumed vaccinations and per capita costs by sub-group**

| **Demo**  **-graphic** | **Group** | **Location** | **# of vaccine**  **booking**  **tool visits*** | **# assumed**  **vaccinations**  **(10%)**** | **# assumed**  **vaccinations**  **(20%)**** | **Cost per**  **vaccination**  **(10%)**** | **Cost per**  **vaccination**  **(20%)**** |
| --- | --- | --- | --- | --- | --- | --- | --- |
| All | Total |  | 8068.5 | 806.8 | 1613.7 | 8 | 4 |
| Location | Location | Berlin | 1328.3 | 132.8 | 265.7 | 12.6 | 6.3 |
|  | Location | Germany | 6740.2 | 674 | 1348 | 7.1 | 3.6 |
| Age | 18-24 | Berlin | 101.8 | 10.2 | 20.4 | 13.4 | 6.7 |
|  | 25-34 | Berlin | 441.5 | 44.2 | 88.3 | 12.9 | 6.5 |
|  | 35-44 | Berlin | 332.7 | 33.3 | 66.5 | 12 | 6 |
|  | 45-54 | Berlin | 225 | 22.5 | 45 | 12.6 | 6.3 |
|  | 55-64 | Berlin | 132.3 | 13.2 | 26.5 | 12.7 | 6.3 |
|  | 65+ | Berlin | 94.8 | 9.5 | 19 | 11.9 | 6 |
|  | 18-24 | Germany | 219.2 | 21.9 | 43.8 | 5.8 | 2.9 |
|  | 25-34 | Germany | 1311.2 | 131.1 | 262.2 | 5.7 | 2.8 |
|  | 35-44 | Germany | 1607.5 | 160.7 | 321.5 | 6.2 | 3.1 |
|  | 45-54 | Germany | 1534.1 | 153.4 | 306.8 | 7.2 | 3.6 |
|  | 55-64 | Germany | 1169.2 | 116.9 | 233.8 | 8.3 | 4.1 |
|  | 65+ | Germany | 899.1 | 89.9 | 179.8 | 9.4 | 4.7 |
| Sex | female | Berlin | 383.7 | 38.4 | 76.7 | 13.9 | 6.9 |
|  | male | Berlin | 939.3 | 93.9 | 187.9 | 12 | 6 |
|  | unknown | Berlin | 5.4 | 0.5 | 1.1 | 12.6 | 6.3 |
|  | female | Germany | 2488.9 | 248.9 | 497.8 | 8.5 | 4.2 |
|  | male | Germany | 4227.7 | 422.8 | 845.5 | 6.3 | 3.1 |
|  | unknown | Germany | 23.6 | 2.4 | 4.7 | 7.7 | 3.9 |
| Target Group | Arabic | Berlin | 1328.3 | 132.8 | 265.7 | 12.6 | 6.3 |
|  | Arabic | Germany | 3480.2 | 348 | 696 | 4.5 | 2.3 |
|  | Russian | Germany | 1281.2 | 128.1 | 256.2 | 12.5 | 6.3 |
|  | Turkish | Germany | 1978.8 | 197.9 | 395.8 | 8.1 | 4.1 |

Note: Data collected by authors, November 2021.

*On average, 53.6% of users in Berlin who clicked on the advertisement (see Table S1) also arrived at the vaccine appointment booking section on our website created for this study. This percentage was then applied to the sample of Germany where tracking was not possible (see Methods section).

** We applied two scenarios assuming that 10% to 20% of Facebook users who visited the vaccine booking tool eventually received the vaccination. The scenarios is based on comparable industry benchmarks for conversion rates (see Methods). Costs per vaccination were calculated based on the total advertisement cost (6,445 €) over the number of estimated vaccinations for each scenario.

Literature Cited

1. Statistisches Bundesamt. Bevölkerung und Erwerbstätigkeit - Bevölkerung mit Migrationshintergrund: Ergebnisse des Mikrozensus 2020. Reihe 2.2. Wiesbaden: DESTATIS; 2020. (Fachserie 1).

2. Tankwanchi AS, Jaca A, Larson HJ, Wiysonge CS, Vermund SH. Taking stock of vaccine hesitancy among migrants: a scoping review protocol. BMJ Open 2020; 10(5):e035225.

3. Robertson E, Reeve KS, Niedzwiedz CL, Moore J, Blake M, Green M et al. Predictors of COVID-19 vaccine hesitancy in the UK household longitudinal study. Brain Behav Immun 2021; 94:41–50.

4. Kamal A, Hodson A, Pearce JM. A Rapid Systematic Review of Factors Influencing COVID-19 Vaccination Uptake in Minority Ethnic Groups in the UK. Vaccines (Basel) 2021; 9(10).

5. Waterman LZ. Cultural competence in vaccine rollout: migrants would face difficulties accessing covid-19 vaccines. BMJ 2021; 372:n220.

6. van Tubergen F, Sindradóttir J. The Religiosity of Immigrants in Europe: A Cross-National Study. Journal for the Scientific Study of Religion 2011; 50(2):272–88.

7. BAMF. Die Religionszugehörigkeit, religiöse Praxis und soziale Einbindung von Geflüchteten. Nürnberg: BAMF; 2020. (BAMF-Kurzanalyse).

8. Henry-Huthmacher C, Hoffmann E. Eltern mit Zuwanderungsgeschichte gewinnen; 2021.

9. Schaeffer, D., & Horn, A., Evaluation der Patienteninformation und -beratung für türkisch- und russischsprachige Migrantinnen und Migranten; 2013. (Veröffentlichungsreihe des Instituts für Pflegewissenschaft an der Universität Bielefeld, no.P13-150, Bielefeld: Institut für Pflegewissenschaft an der Universität Bielefeld.).

10. Orazi DC, Johnston AC. Running field experiments using Facebook split test. J Bus Res 2020; 118:189–98.

11. Matz SC, Kosinski M, Nave G, Stillwell DJ. Psychological targeting as an effective approach to digital mass persuasion. Proc Natl Acad Sci U S A 2017; 114(48):12714–9.

12. Irvine M. Facebook Ad Benchmarks for your industry; 2022. Available from: URL: https://www.wordstream.com/blog/ws/2017/02/28/facebook-advertising-benchmarks.

1. Estimated Facebook audience size in Germany according to Facebook's ad platform (as of 24 January 2022). Available at: https://business.facebook.com/adsmanager/ [↑](#footnote-ref-1)
